# Evaluating Manganese-Doped Magnetic Nanoflowers for Biocompatibility and In Vitro Magnetic Hyperthermia Efficacy

**DOI:** 10.3390/pharmaceutics17030384

**Published:** 2025-03-18

**Authors:** Andreea-Elena Petru, Cristian Iacovita, Ionel Fizeșan, Roxana Dudric, Ionut-Valentin Crestin, Constantin Mihai Lucaciu, Felicia Loghin, Bela Kiss

**Affiliations:** 1 Department of Toxicology, Faculty of Pharmacy, “Iuliu Hațieganu” University of Medicine and Pharmacy, Pasteur 6A, 400349 Cluj-Napoca, Romania; andreea.elen.petru@elearn.umfcluj.ro (A.-E.P.); ionel.fizesan@umfcluj.ro (I.F.); ionut.vale.crestin@elearn.umfcluj.ro (I.-V.C.); floghin@umfcluj.ro (F.L.); kbela@umfcluj.ro (B.K.); 2 Department of Pharmaceutical Physics-Biophysics, Faculty of Pharmacy, “Iuliu Hatieganu” University of Medicine and Pharmacy, Pasteur 6, 400349 Cluj-Napoca, Romania; cristian.iacovita@umfcluj.ro; 3 Faculty of Physics, “Babes Bolyai” University, Kogalniceanu 1, 400084 Cluj-Napoca, Romania; roxana.pacurariu@phys.ubbcluj.ro

**Keywords:** nanoflowers, manganese-doped iron oxide nanoparticles, magnetic hyperthermia, cancer therapy, A549 cancer cell line, in vitro MH

## Abstract

**Background/Objectives**: Magnetic hyperthermia (MH) has emerged as a promising alternative to conventional cancer treatments, offering targeted tumor destruction with minimal damage to healthy tissues. In this study, we synthesized manganese-doped magnetic nanoflowers (Mn-NFs) using a polyol-mediated approach to enhance heating efficiency and biocompatibility for MH applications. Our objective was to evaluate their structural, magnetic, and in vitro hyperthermic properties to determine their potential for lung cancer therapy. **Methods**: Mn-NFs, with the general formula MnxFe_3_-xO_4_ (x = 0, 0.3, 0.5, 0.7), were synthesized via a one-step polyol method and characterized using transmission electron microscopy (TEM), X-ray diffraction (XRD), and vibrating sample magnetometry (VSM). Their heating efficiency was assessed through specific absorption rate (SAR) measurements in aqueous and solid environments under an alternating magnetic field (AMF). Cytocompatibility was evaluated using the Alamar Blue assay on A549 lung carcinoma cells. Cellular uptake was quantified via a colorimetric iron determination method, while in vitro MH efficacy was tested by subjecting Mn-NF-loaded A549 cells to AMF exposure at different field strengths and nanoparticle concentrations. **Results**: Mn-NFs exhibited a flower-like morphology with enhanced magnetic properties, achieving high SAR values, particularly in immobilized conditions. Cytotoxicity assays confirmed high biocompatibility at relevant doses, with Mn-NFs of x = 0.3 showing optimal cellular uptake. MH studies demonstrated significant cancer cell death at AMF intensities of around 30 kA/m, with increased effectiveness following static magnetic field pre-alignment. **Conclusions**: The results highlight Mn-NFs, particularly those with a Mn content of x = 0.3, as promising candidates for MH-based lung cancer therapy, combining high heating efficiency, biocompatibility, and effective intracellular uptake. Further studies are needed to validate their therapeutic potential in vivo.

## 1. Introduction

Conventional cancer treatments, such as chemotherapy and radiotherapy, can be effective but often cause significant damage to healthy tissues, leading to a decline in the patient’s overall condition [1,2]. This has created a growing need for more targeted and less harmful treatment options. One promising approach is magnetic hyperthermia (MH), which uses the heat generated by magnetic nanoparticles (MNPs) when exposed to an alternating magnetic field (AMF) [3,4]. This method is noninvasive and has no limits on how deep the magnetic field can penetrate the body. MH works by exploiting the fact that cancer cells are more sensitive to heat than healthy cells. When the temperature rises above 42 °C, tumoral growth is inhibited, while healthy cells can tolerate the higher heat [5,6].

In magnetic hyperthermia (MH), the key parameters of the alternating magnetic field (AMF) are frequency (f) and magnetic field amplitude (H). The safety limit for their product (H × f) has recently been increased from 5 × 10^9^ A/m∙s to 9.8 × 10^9^ A/m∙s [7,8]. As a result, the effectiveness of MH treatment depends heavily on the intrinsic properties of MNPs, including saturation magnetization (M_s_), coercivity (H_c_), and anisotropy (K) [9,10]. For biomedical applications, MNPs must also exhibit a superparamagnetic (SP) state at room temperature, allowing their net magnetization to be controlled by a weak external magnetic field, which helps prevent particle agglomeration [11,12]. However, spherical SP iron oxide nanoparticles, called SPIONs, have low M_s_ and release insufficient heat under AMF, reducing MH treatment efficiency [13,14]. Therefore, it is crucial to use MNPs with augmented magnetization per particle.

Extensive efforts have been directed toward developing MNPs with significantly higher specific absorption rate (SAR) values than conventional SPIONs. Two primary strategies have emerged: one focusing on single-core MNPs [15,16] and the other on SPION assemblies [17,18]. Increasing the size of SPIONs can shift them into a ferrimagnetic (FM) state, resulting in SAR values being nearly ten times higher [19,20,21]. However, this comes at the cost of reduced colloidal stability, as dipole–dipole interactions cause clustering [22]. Among anisotropic SPIONs, cubic SPIONs have shown a notable increase in SAR values due to their low surface anisotropy, which is linked to fewer disordered spins [23,24,25]. Additionally, incorporating zinc (Zn^2+^) and manganese (Mn^2+^) cations into the tetrahedral sites of the magnetite spinel structure significantly enhances the saturation magnetization (M_s_), further increasing SAR compared to pure magnetite [26,27,28,29]. Recently, iron-based nano-rings have emerged as excellent candidates for both in vitro and in vivo MH applications [30]. The nanoring morphology is associated with a magnetic vortex signature, displaying the hysteresis loop collapsing in the low magnetic field range, reducing thus their remanent magnetization and dipolar interaction, which is the main cause for particle agglomeration.

In recent years, clustering SPIONs into controlled aggregates has become a promising alternative for creating magnetic entities with superior heating capabilities [31,32,33]. Multicore iron oxide nanoparticles (NPs), known as nanoflowers (NFs), are particularly promising for magnetic hyperthermia (MH) [34,35,36,37,38]. NFs offer several advantages over single-core SPIONs, including the following: (1) enhanced magnetic properties: their interconnected cores exhibit a superferromagnetic state, leading to a higher overall magnetic moment and increased M_s_, improving responsiveness to external magnetic fields [39,40,41]; (2) improved stability: the interconnected structure resists aggregation, maintaining magnetic properties over time and under various environmental conditions; (3) larger surface area: this facilitates more efficient interactions with target molecules or surfaces, making NFs ideal for applications such as drug delivery, magnetic separation, and catalysis; (4) multiple binding sites: the multi-core structure provides numerous attachment points, enhancing binding efficiency and the capture or immobilization of target substances.

Multicore iron oxide NPs are typically synthesized using either a two-step method [42,43,44], where pre-synthesized magnetic nanocrystals are assembled into clusters with a specific configuration, or a one-step method, where the self-assembly process occurs simultaneously with nanocrystal formation. For biomedical applications, which require time-efficient, cost-effective, scalable, and highly reproducible MNPs, the one-step synthesis methods—commonly referred to as one-pot processes—are generally preferred. Among these, the polyol synthesis method using ethylene glycol (EG) as a solvent has proven particularly effective. It promotes the formation of clusters with higher crystallinity (and thus higher M_s_) while allowing precise control over the size of the aggregates, which can easily be dispersed in aqueous media [45,46,47].

In this study, we synthesized multicore iron oxide nanoparticles (NPs) with a flower-like architecture using the polyol-mediated method. This approach was conducted at a temperature well above the boiling point of ethylene glycol (EG), reducing the reaction time required to form nanoflowers (NFs) to just one hour [48,49]. The elevated temperature produced magnetic nanoparticles (MNPs) with high crystallinity, driving them into a ferrimagnetic (FM) state. Aiming to enhance their magnetic softness and increase their M_s_, we doped the NFs with manganese [50,51,52]. The NFs with the best heating performance were selected for further analysis, including toxicity testing and in vitro magnetic hyperthermia evaluation on a human lung cancer cell line (A549).

## 2. Materials and Methods

### 2.1. Synthesis

MNPs were synthesized by using the following products (of analytical grade and without any further purification): iron(III) chloride hexahydrate (FeCl_3_∙6H_2_O) ≥ 98%, manganese(II) acetylacetonate [Mn(acac)_2_] ≥ 98%, ethylene glycol (EG) ≥ 99%, and sodium acetate trihydrate (NaAc∙3H_2_O) ≥ 99.5%, purchased from Carl Roth GmbH, Karlsruhe, Germany.

NFs were prepared using the conventional polyol-mediated synthetic route [45]. The reaction mixture was prepared as follows: 3.9 mmol of FeCl_3_∙6H_2_O together with 1.1 g of NaAc∙3H_2_O was dissolved in 55 mL of EG by magnetic stirring for 1 h at a temperature of 50 °C. In the case of Mn-doped NFs, different FeCl_3_∙6H_2_O/Mn(acac)_2_ ratios in mmol have been used to obtain Mn ferrites with the general formula Mn_x_Fe_3–x_O_4_, as follows: 3.47/0.43, 3.25/0.65, and 2.975/0.925 to obtain x = 0.3, 0.5, and 0.7, respectively. The mixtures were transferred to a glass vessel, placed inside a stainless-steel container, and then subjected to nitrogen bubbling (5 min) to eliminate oxygen (dissolved in the solvent or adsorbed on the suspended reagent particles). After this, the stainless-steel container was tightly sealed with a Teflon gasket and five screws. The assembly was placed in an oven and heated to 300 °C with a temperature ramp of 10 °C/min at atmospheric pressure. The mixture was maintained at this temperature for 1 h, then allowed to cool to room temperature gradually. After cooling, the NFs from the mixtures were separated using a neodymium magnet, and the supernatant was mechanically removed. To remove residual reaction products, the obtained sediments were redispersed in a double-distilled water-ethanol mixture (volume ratio ~1:1) and then decanted with the help of the magnet. This process was repeated several times. Finally, the sediments were dispersed in ~20 mL of double-distilled water and stored in plastic containers.

### 2.2. Characterization

The morphology and size of NFs were evaluated in a JEOL JEM-100CX II (JEOL, Tokyo, Japan) transmission electron microscope, operating at 80 kV, equipped with a MegaView G3 camera (Emsis, Münster, Germany) running with Radius 2.1 software (Emsis). Samples were prepared by placing one drop of water suspension of NFs (10 µg_MNPs_/mL) on a carbon-coated copper grid. After 5 min, the excess water was removed with filter paper. The size distributions of MNPs were determined through a manual measurement of around 500 MNPs for each sample using ImageJ software Version 1.54k (U. S. National Institutes of Health, Bethesda, MD, USA, https://imagej.net/ij/, accessed on 11 November 2024). The histograms were obtained using the OriginPro 2022 Academic software (OriginLab Corporation, Northampton, MA, USA) and fitted with a log-normal distribution using the same software.

The crystalline structure of NFs was determined by X-ray powder diffraction conducted in a Bruker D8 Advance diffractometer using Cu Kα radiation (Bruker AXS GmbH, Karlsruhe, Germany).

Magnetic measurements at 4 K and 300 K were carried out in a vibrating sample magnetometer (VSM) device from Cryogenic Limited (London, UK) under applied magnetic fields from 0 to ± 4 T.

The heating efficiency was evaluated using a commercially available magnetic hyperthermia system, the Easy Heat 0224 from Ambrell (Scottsville, NY, USA) equipped with an optical fiber temperature sensor (0.1 °C accuracy). A volume of 0.5 mL of MNPs was placed in the center of an 8-turn coil using a thermally isolated Teflon holder and then submitted to an AC magnetic field with a fixed frequency (355 kHz) and variable amplitude (10–60 kA/m). Details of specific absorption rate (SAR) calculations are provided in the Supplementary Materials (Section S1).

### 2.3. Determination of Iron Content via Liebig Reaction

The iron content of NFs was measured using the Liebig reaction. In this regard, approximately 5 mg of NFs was magnetically separated and further mixed with 10 mL of 12% HCl solution. The digestion was performed for 6 h at 80 °C, and the obtained solutions were centrifuged at 12,000× *g* for 10 min to obtain the supernatants. The total Fe^3+^ content of supernatants (50 µL) was measured after an oxidation step with 1% ammonium persulfate (50 µL) by the reaction with 0.1 M potassium thiocyanate (100 µL) that yields a red-colored iron–thiocyanate complex. The absorbance was measured at λ = 490 nm using a Synergy 2 Multi-Mode Microplate Reader (Agilent Bio Tek, Santa Clara, CA, USA), and the Fe^3+^ content was calculated from a standard curve with concentrations ranging between 5 and 140 µg/mL (Supplementary Materials—Section S2) [48].

### 2.4. Evaluation of Optical Interference of Mn in the Determination of Iron Content via Liebig Reaction

Given the method employed to measure the iron content of NFs and that three of the four types of NFs that were synthesized were further doped with manganese, the issue was whether Mn, a transitional metal, could impede or interfere with the Liebig reaction. Thus, the Liebig reaction was performed in the presence and absence of Mn^2+^ ions. Briefly, solutions of Fe^3+^ with concentrations between 3.06 and 200 µg/mL and Mn^2+^ solutions with concentrations between 25 and 400 µg/mL were prepared. Standard curves for iron determination (3.06–200 µg/mL) were employed, as previously described in the Supplementary Materials, Section S2, in the presence of Mn^2+^ for each concentration (25 µg/mL, 50 µg/mL, 100 µg/mL, 200 µg/mL, 400 µg/mL). The red-colored iron–thiocyanate complex was quantified measuring the absorbance at λ = 490 nm using a Synergy 2 Multi-Mode Microplate Reader. Based on Supplementary Materials, Section S3, no interference of Mn^2+^ was observed. The absorbance values were constant, regardless of Mn^2+^ concentration, indicating that Mn^2+^ did not interfere and did not impede the reaction with Fe^3+^ ions.

### 2.5. Cell Lines

The human pulmonary carcinoma A549 cell line, purchased from the American Type Culture Collection (ATCC, Manassas, VA, USA), was employed as a model in the current research. All the following cell culture reagents were purchased from Gibco, Paisley, UK: Dulbecco’s Modified Eagle’s Medium (DMEM), Fetal Bovine Serum (FBS), Phosphate Buffer Saline (PBS), Trypsin-EDTA, and Penicillin/Streptomycin (10,000 U/mL). Typically, cell cultures were maintained in an incubator with 5% CO_2_ at a temperature of 37 °C. On alternate days, the cellular medium was refreshed. The cells were either sub-cultured or used in further experiments, once the confluency was reached (80–100%).

### 2.6. In Vitro Cytocompatibility Evaluation

The cytotoxicity assessment of NFs was performed by employing the Alamar Blue (AB) assay on cancerous cells. The Alamar Blue assay measures cellular respiration by detecting the conversion of resazurin to resorufin by viable cells. It is useful for checking the viability of cell cultures because it is a non-toxic, with high sensitivity and a dynamic range, being also easy to use, compatible with various cell types. Briefly, cells were seeded in 12-well plates and left to attach overnight. Subsequently, the cells were treated with different concentrations of MNPs, as follows: 15.625, 31.25, 62.5, 125, and 250 µg/cm^2^. Following the treatment, cells were thoroughly washed with PBS and incubated with the AB reagent. The AB reagents consist of a 200 µM resazurin solution. The metabolically active cell-dependent conversion of resazurin to resorufin was determined by measuring the fluorescence at an λ_excitation_ = 530/25 nm and λ_emission_ = 590/35 nm. Cells exposed to cellular media without NFs served as negative controls for data normalization. All the experiments were conducted in three biological replicates and fluorescence measurement was performed using the Synergy 2 Multi-Mode Microplate Reader.

### 2.7. Evaluation of Cellular Uptake Using Prussian Blue Staining and Liebig Reaction

The experiment was conducted in the same conditions as the one for the in vitro cytotoxicity assessment (12-well plates, the same number of cells seeded as for the viability tests, 24 h incubation). For the qualitative evaluation of the cellular uptake, Prussian Blue staining was employed, as previously described [48]. The cells were fixed with 4% paraformaldehyde for 30 min at room temperature and stained with a mixture containing HCl 2% with 2% potassium ferrocyanide aqueous solutions. Once the intracellular iron was stained, the cells were washed three times with PBS and counterstained with Eosin. Finally, the cells were visualized using a light microscope at a magnification of 100× and representative images were taken using a photo camera. For the quantitative determination, the cells were washed two times with PBS, trypsinized, and centrifuged at 4500× *g* for 5 min. The internalized iron was quantified employing the Liebig reaction of free Fe^3+^ with thiocyanate in an acidic medium, as mentioned in the Supplementary Materials (Section S2) [48].

### 2.8. In Vitro Magnetic Hyperthermia

For in vitro MH studies, around 1 × 10^6^ A549 cells were cultivated in 6-well plates. After being left to attach overnight, the cells were exposed to non-toxic doses of NFs for 24 h (cellular viability higher than 80%). The other day, the excess NFs were removed, while the attached cells with the internalized NFs were detached using trypsin-EDTA. The cellular suspension obtained after trypsinization was equally divided into four aliquots of 1500 μL each, following the centrifugation and the removal of 1200 μL of cellular media. The obtained four pellets composed of cells with internalized MNPs and dispersed in 300 μL of cellular media were further used for in vitro MH. Before MH treatment, two pellets were exposed for 30 s to a static magnetic field of 65 kA/m strength (Supplementary Materials—Section S4). From the total of four pellets, two of them were exposed to an AMF (different amplitude and constant frequency) for 30 min with the system stabilized beforehand at 37 °C, while the other two pellets serving as a negative control were maintained at 37 °C in a thermostatic water bath. Post-MH, the cells were seeded in 96-well plates in 6 technical replicates and left to attach for 24 h. Subsequently, an AB viability test was conducted as previously described (Section 2.6). The efficacy of MH treatment was evaluated based on the cellular viability obtained by employing AB.

### 2.9. Statistics

The results are expressed as average values ± standard deviation (SD). Data analysis and graphical representation were performed in SigmaPlot 11.0 computer software (Systat Software Inc., Chicago, IL, USA) and GraphPad Prism 7.00 (GraphPad Software, San Diego, CA, USA).

## 3. Results and Discussion

### 3.1. Structural Characterization of NFs

**Figure 1:**
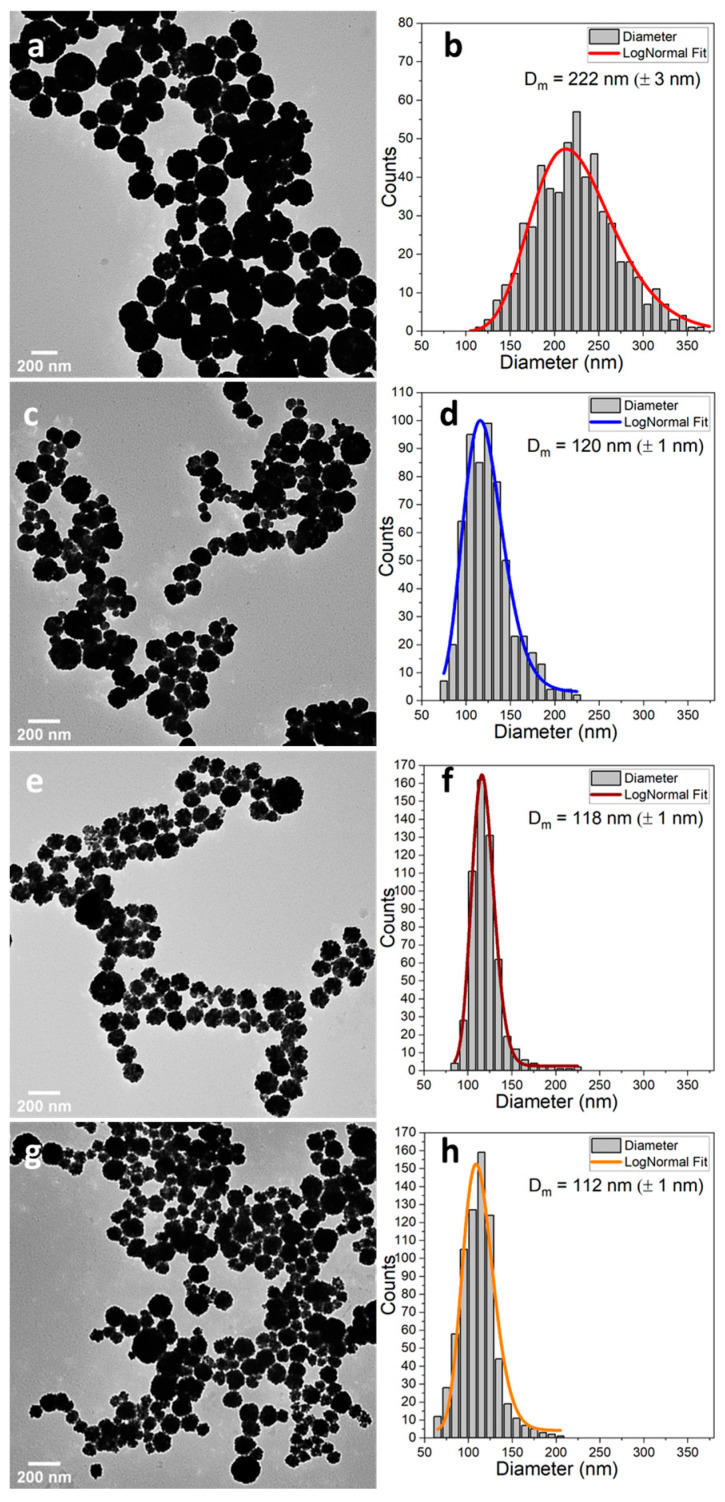
Large-scale TEM images and their corresponding size distribution histograms fitted to a log-normal distribution of (**a**,**b**) Mn00, (**c**,**d**) Mn03, (**e**,**f**) Mn05, and (**g**,**h**) Mn07 MNPs.

Employing the polyol synthesis method under high temperature and pressure, a series of ferrite NFs of the type Mn_x_Fe_3−x_O_4_ (where 0 ≤ x ≤ 0.7) were synthesized in a short time. Ethylene glycol was used as the solvent, with sodium acetate serving as the reducing agent. Initially, no Mn(acac)_2_ precursor was added to the reaction mixture, resulting in MNPs (referred to as Mn00) that exhibited a spherical nanocluster morphology with an average diameter of 222 nm (±3 nm) (Figure 1a,b). The size distribution was broad, with a standard deviation (σ) of 49.13 nm (±4 nm) (Figure 1b).

Subsequently, FeCl_3_∙6H_2_O to Mn(acac)_2_ molar ratios of 0.3, 0.5, and 0.7 were employed in the synthesis to produce Mn-doped ferrite (denoted Mn03, Mn05, Mn07) and to investigate the effect of the Mn^2+^ cation on the properties of the resulting MNPs. The introduction of Mn into the reaction mixture modified the MNP morphology from nanocluster to nanoflower in all three cases (Figure 1c,e,g). This addition also significantly reduced the mean diameter to 120 nm (±1 nm), 118 nm (±1 nm), and 112 nm (±1 nm) for Mn03, Mn05, and Mn07, respectively (Figure 1d,f,h). The size distributions became narrower, with standard deviations decreasing to 23.21 nm (±2 nm), 12.67 nm (±1 nm), and 17.81 nm (±2 nm) for Mn03, Mn05, and Mn07, respectively (Table 1).

**Figure 2:**
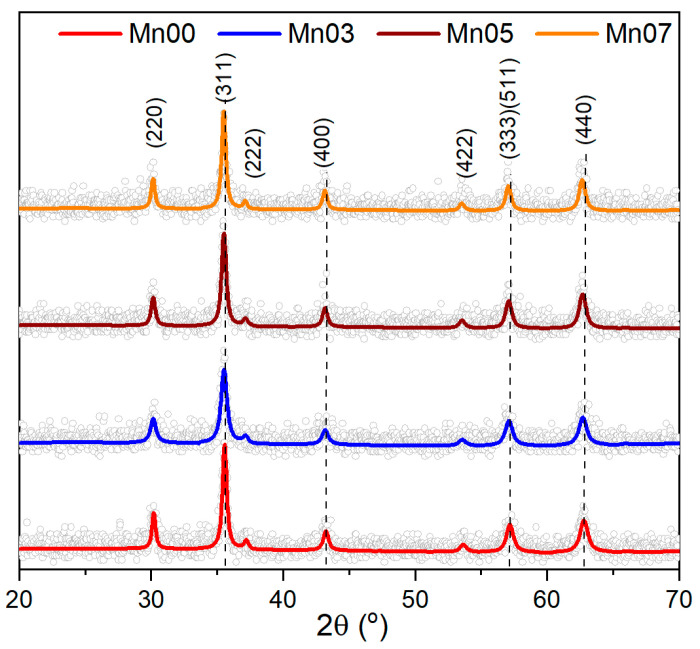
XRD diffraction patterns of all four types of MNPs. The dashed black lines indicate the progressive shift of all diffraction peaks towards lower angles. The diffractograms were shifted for clarity.

To gain deeper insight into the crystalline structure of the MNPs, X-ray diffraction (XRD) analysis was conducted on the powder samples. As depicted in Figure 2, the XRD pattern distinctly revealed the presence of a pure inverse spinel crystalline structure in the Mn00 sample. The positions and relative intensities of all diffraction peaks corresponded to magnetite Fe_3_O_4_ (PDF number: 88-0315). Notably, no peaks associated with FeO or Fe_2_O_3_ were observed, confirming that the MNPs are composed solely of pure magnetite Fe_3_O_4_. The black color of the powder further substantiated the purity of the magnetite phase. The lattice parameters (a = 8.379 Å) were closely aligned with those of bulk magnetite (a = 8.375 Å). The average crystalline size of the Mn00 sample, determined from the (220), (311), and (440) diffraction peaks using Scherrer’s formula, was 33.06 nm (±5 nm). This value is much smaller than the average diameter observed in TEM images, indicating the multicore nature of the large spherical Mn00 NPs.

The XRD patterns of the three Mn-doped samples were identical to that of the Mn00 sample (pure Fe_3_O_4_), confirming the presence of magnetite across all diffractograms (Figure 2). The XRD peaks indicated a single-phase cubic spinel crystalline structure, with no additional peaks corresponding to manganese oxides or hydroxides (Figure 2). As the composition varied from Mn00 to Mn07, the XRD peaks progressively shifted towards lower angles, suggesting the incorporation of Mn^2+^ into the crystalline structure [53]. The inclusion of Mn^2+^ expanded the crystalline structure of MNPs, resulting in increased lattice parameters: 8.384 Å, 8.397 Å, and 8.424 Å for Mn03, Mn05, and Mn07, respectively. Compared to the Mn00 sample, the average crystalline size decreased to 21 nm (±1 nm), 23 nm (±1 nm), and 24 nm (±2 nm) for Mn03, Mn05, and Mn07, respectively (Table 1).

### 3.2. Magnetic Characterization of NFs

**Figure 3:**
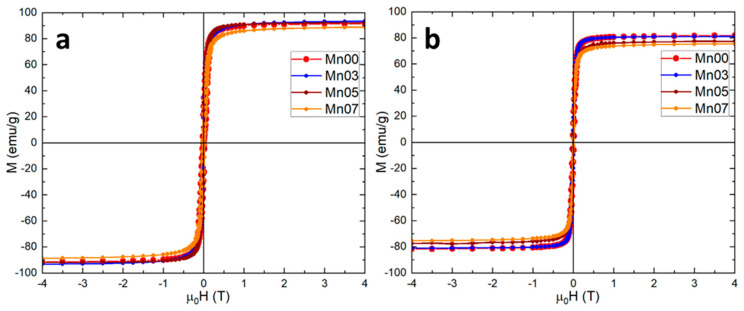
Hysteresis loops of all four types of MNPs at (**a**) 4 K and (**b**) 300K.

The magnetic properties of all four samples were further investigated by measuring hysteresis loops at both 4 K and 300 K (Figure 3). The MNPs displayed ferrimagnetic behavior at 4 K, which persisted up to 300 K, retaining both remanence and coercivity (Supplementary Materials—Section S5). The saturation magnetization (M_s_) for the Mn00 sample of 91.5 emu/g closely matched that of bulk magnetite (92 emu/g), reaffirming the pure magnetite nature of the nanoclusters. Across the Mn/Fe ratios studied, the M_s_ slightly increased to 93.1 emu/g and 92 emu/g for the Mn03 and Mn05 samples, respectively, while a slight decrease to 88.5 emu/g was observed for the Mn07 sample (Table 2). The behavior is attributed to the degree of inversion of the spinel structure; for an inverse spinel, the M_s_ increases with a higher Mn/Fe ratio, whereas for a normal spinel, the M_s_ decreases [54]. At room temperature, M_s_ values generally decreased, with the Mn00 sample showing a reduction of only 10 emu/g, while the Mn-doped MNPs experienced a drop of 12–15 emu/g (Table 2). Despite this, the high M_s_ values at room temperature suggest that all four types of MNPs are likely to exhibit significant SAR values. The highest coercive field (H_c_) at 4 K was observed in the Mn00 sample (Fe_3_O_4_), while Mn-doped NFs showed a slight decrease in H_c_ with increasing Mn/Fe ratios, consistent with the known softness of manganese ferrites compared to magnetite (Table 2) [55]. This trend in H_c_ persisted at room temperature, although the values were nearly halved (Table 2).

For the Mn00 sample, the zero-field-cooled (ZFC) and field-cooled (FC) magnetization curves remain separated at 300 K, suggesting significant dipolar interactions among the MNPs, which tend to raise the blocking temperature (Supplementary Materials—Section S6). However, for the Mn-doped samples, the ZFC and FC curves converge below 300 K. The blocking temperature decreases from 295 K to 270 K as the Mn doping level increases from Mn03 to Mn07, indicating a reduction in dipolar interactions and an increase in the softness of the magnetic phase with higher Mn doping [55].

### 3.3. Magnetic Hyperthermia Properties of NFs

**Figure 4:**
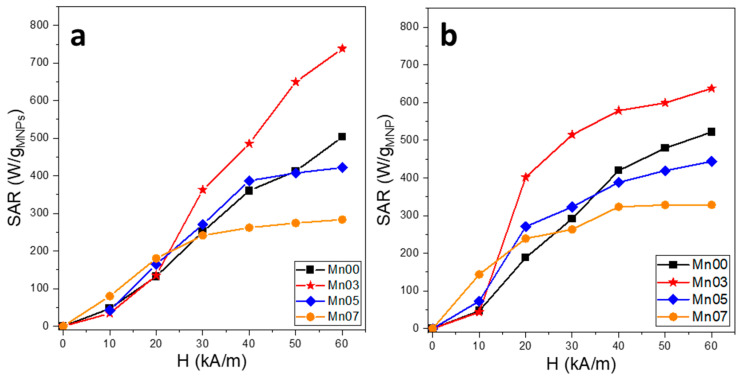
SAR values of all four types of NFs dispersed in (**a**) water and (**b**) PEG8k at a concentration of 1 mg_MNPs_/mL.

The calorimetric method was employed to evaluate the heating performance of NFs. A quantity of 1 mg of NFs was dispersed in either 1 mL of water or embedded within a solid polyethyleneglycol matrix (PEG8k) to record the heating curves. In the second scenario, the NFs were mixed with melted PEG8K at approximately 80 °C. The magnetic field amplitude (H) was adjusted from 10 kA/m to 60 kA/m in increments of 10 kA/m, while the frequency was maintained at 355 kHz. The SAR values as a function of H are presented in Figure 4, which, in water, shows two distinct trends. For the Mn00 and Mn03 samples, the SAR values increase progressively with increasing H, reaching, at the highest H value, 500 W/g_MNPs_ and 740 W/g_MNPs_, respectively. In contrast, the SAR values for the Mn05 and Mn07 samples reach a saturation level (around 400 W/g_MNPs_ and 275 W/g_MNP_s, respectively) beginning at H of 40 kA/m. At the first two H values (10 kA/m and 20 kA/m), which are close to the H_c_ values of the NFs, the SAR values for all four samples are quite similar, with Mn07 NFs showing a slightly higher SAR, probably due to its higher magnetic softness character. Starting at 30 kA/m, Mn03 exhibits a superior SAR value compared to the other NFs, with the gap widening as H increases.

The heating properties of MNPs in a ferrimagnetic state, as in our case, can be explained using models based on the Stoner-Wohlfarth theory, based on their dynamic hysteresis curve, that can be computed numerically, with the area of the hysteresis loop representing the heat generated by the MNPs during a single cycle. However, there is currently no analytical function derived from a theoretical model in the literature that links the magnetic properties of MNPs to their specific absorption rate (SAR) performance [56].

Hysteresis losses depend on the intensity of the applied magnetic field H, the coercive field of the MNP assembly, and their magnetization. For low values of H, the hysteresis area is very small, and consequently, the SAR values are insignificant. For H values greater than the coercive field, the hysteresis area becomes larger as H increases, resulting in a steep increase in the SAR values up to a saturation value. The further increase in H will lead to a plateau of the SAR values [57]. However, in the case of the Mn00 and Mn03 samples, the saturation is less obvious. This behavior might be explained by a much larger size distribution of MNPs in these latter samples.

The MH experiments on NFs dispersed in water do not represent a realistic determination of their heating performances in both in vitro and in vivo conditions. In water, due to magnetic interactions and their mobility, the MNPs can physically rotate, self-organize, or form chains or columns along the magnetic field direction (under the influence of the field used in MH), changing the effective anisotropy and magnetization of the MNP dispersion and thus their dynamic hysteresis area. It has been proven that this self-organization in a liquid environment can significantly enhance the MNP’s heating power as compared to MNPs immobilized in a cellular environment [58,59].

From another point of view, in water, both Néel and Brown relaxation mechanisms can occur and the MNP heating capabilities under an external alternating magnetic field are different compared to environments where their physical rotation is blocked. Therefore, to more accurately simulate both in vitro and in vivo conditions, where MNPs tend to become immobilized and form aggregates in cells [60,61], a second set of MH experiments has been performed, focused on NFs dispersed within a solid matrix of PEG8K. In the liquid state, before solidification, the samples were exposed to a static magnetic field with a strength of approximately 65 kA/m, which caused the MNPs to align, at least with their easy magnetization axis parallel to the lines of the AMF in the MH setup (Supplementary Materials—Section S4). Our previous results and several other studies pointed out this effect of increasing the SAR by the pre-alignment of the MNPs before being immobilized (gelled) [57,62].

We would expect the pre-alignment of the MNPs before the immobilization to restore the SAR measured in water. Between 10 and 40 kA/m, the SAR of all four types of NFs dispersed in PEG8k increases with H, while for a higher H, it shows a slight tendency to saturate. Compared to the SAR recorded in water, even though the NF particles are immobilized in a solid matrix, they exhibit higher SAR values (Supplementary Materials—Section S7). This indicates that in water, the NFs behave differently compared to small-size MNPs as they tend to aggregate rapidly, forming large aggregates that cannot be controlled by the strength of AMF. The NFs in the Mn00, Mn05, and Mn07 samples show superior SAR values in PEG8k compared to those in water, across the entire range of H (Figure 4). However, the NFs in the Mn03 sample exhibit a different behavior in the solid matrix. Firstly, it is notable that these NFs display the largest difference in SAR values within the 10–40 kA/m range: for example, at 20 kA/m, the SAR in PEG8k increases more than threefold compared to that in water. However, at high fields of 50 kA/m and 60 kA/m, the SAR in PEG8k is lower than that recorded in water. This demonstrates the influence of an AMF of high amplitudes on the NFs dispersed in water, suggesting that the NFs structure themselves into columns aligned with the AMF lines, resulting in a higher SAR compared to PEG8k, where this structuring is hindered by the high viscosity of the medium.

Within cells, NFs are enclosed in intracellular vesicles, which prevent them from aggregating into large clusters, significantly reducing their heating efficiency, as observed in water. Moreover, dipolar interactions between different NF assemblies within intracellular vesicles are minimized, suggesting that internalized NFs could generate sufficient heat to induce apoptosis in cancer cells. Due to their large size and confinement within intracellular vesicles, NFs have extremely limited movement. This restriction makes it highly improbable for them to align and form large chains under the influence of AMF, a behavior commonly observed in smaller MNPs [63,64]. However, NF-loaded intracellular vesicles may still align along the AMF lines [64]. In this context, exposing NF-loaded cells to a static magnetic field before in vitro MH treatment, as shown in studies conducted in a solid matrix, could enhance the hyperthermic efficiency of NFs. From this perspective, two series of in vitro MH treatments were conducted; in one, the NF-loaded cells were exposed to a static magnetic field beforehand.

### 3.4. In Vitro Cytotoxicity Evaluation of NFs

Given their high potential as heating agents for MH, the synthesized NFs must demonstrate excellent cytocompatibility, meaning they should not harm healthy tissues they encounter. To assess their cytocompatibility, A549 cells (human lung carcinoma epithelial cells) were used in this study, using Alamar Blue (AB) assay as the evaluation method. The first step was to check if the NFs interfered with the optical or biochemical aspects of the viability assay. All synthesized NFs (Mn00, Mn03, Mn05, Mn07) showed no biochemical interference with the assay. For optical interference, a minor issue was observed, but this was easily addressed by adding a centrifugation step and measuring the fluorescent signal in the supernatant [48,64,65].

**Figure 5:**
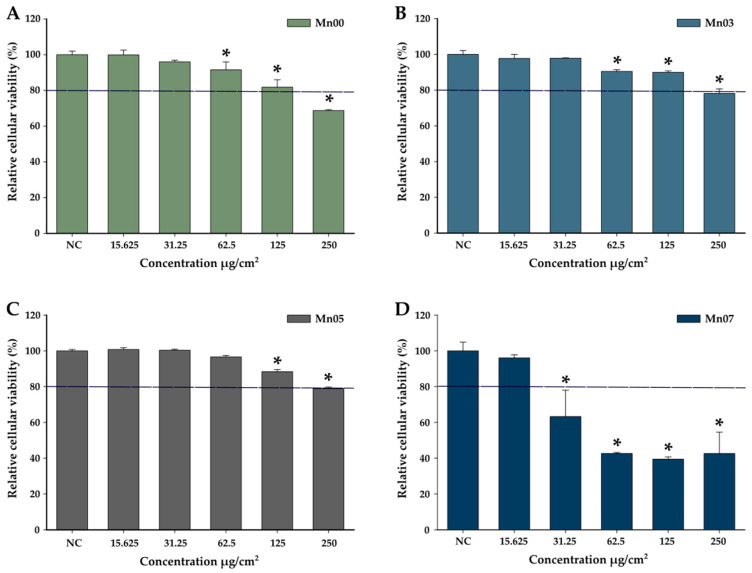
The cytocompatibility of Mn00 (**A**), Mn03 (**B**), Mn05 (**C**), and Mn07 (**D**) on the A549 cell line after a 24 h incubation. Data are presented as relative cellular viability values as compared to the negative control (100%), as mean ± SD of three biological replicates. The significant differences compared to the negative control (ANOVA + Dunn’s; *p* < 0.05) are noted with asterisks (*).

To evaluate the cytocompatibility of the four types of synthesized NFs, cells were exposed to increasing NFs concentrations, ranging from 15.625 µg/cm^2^ to 250 µg/cm^2^, and incubated for 24 h (Figure 5). The type of NFs and their dosage for the in vitro MH experiments were selected based on the AB viability assay results. High biocompatibility is a key factor, as NFs that reduce cellular viability by less than 20% are generally considered safe and non-toxic [48]. When A549 cells were exposed to Mn00 NPs, no significant effect on cellular viability was observed at concentrations between 15.625 µg/cm^2^ and 62.5 µg/cm^2^ (Figure 5A). However, a ~20% reduction in viability was recorded at a concentration of 125 µg/cm^2^, while the highest dose (250 µg/cm^2^) caused a 30% decrease in viability (Figure 5A). In comparison, Mn03 and Mn05 NFs showed good biocompatibility, with cell viability remaining above 80% at all concentrations, except for the highest dose (250 µg/cm^2^), where viability dropped slightly below 80% (Figure 5B,C). Conversely, Mn07 NFs exhibited higher cytotoxicity, with a 40% reduction in cell viability observed at a dose of 31.25 µg/cm^2^ and further decreases at higher concentrations (Figure 5D). The biocompatibility data were fitted with the sigmoidal Hill function (Figure S8), as it was recently demonstrated that it can provide useful insights into the cytotoxicity mechanism of nanoparticles [66]. The main parameters derived from the fittings are presented in Table S1. The LD50, the dose which produces 50% cell death, is highest for Mn03 NFs (735 μg/cm^2^) and is more than one order of magnitude lower in the case of the Mn07 sample (63 μg/cm^2^). The Hill coefficient is close to unity in the case of the most nontoxic samples (Mn03 and Mn05) and slightly higher than 1, indicating a small cooperativity in the case of Mn00 ad Mn07. A study reported in the literature evaluated the Mn doping effect on different properties of CeO_2_ nanoparticles. Of interest for us was the impact of the Mn dopant on the cytocompatibility of the CeO_2_ nanoparticles. Similar to our results, Mn doping did not increase cellular toxicity up to the concentration of 5% Mn content. At the highest concentration, of 7% Mn, the cellular toxicity increased, most probably due to an inability of the cells to cope with the stress induced by ROS generation [67]. The pronounced cytotoxicity of Mn07 NFs could be attributed to the acidic disintegration of Mn ferrites into Mn^2+^, Fe^2+^, and Fe^3+^ ions. The release of Mn^2+^ ions may catalyze the Fenton reaction, generating reactive oxygen species (ROS), which can induce cell death, as reported in the literature [68,69]. Regarding Mn00, Mn03, and Mn05, the recorded cytotoxicities are in line with most of the studies published in the literature, which have shown no/slight cytotoxic effect of Mn ferrites up to an exposure dose of 100–200 μg/ML [70].

### 3.5. Cellular Uptake of NFs

**Figure 6:**
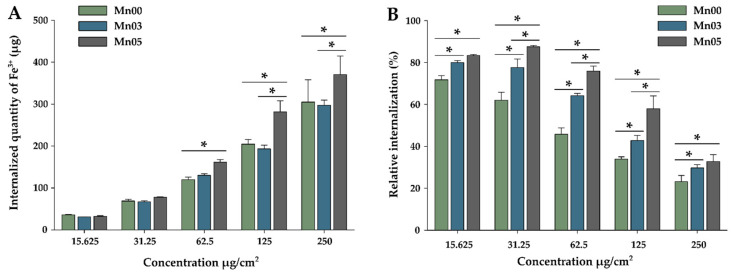
Cellular internalization of NFs in A549 cells after a 24 h exposure: (**A**) total iron amount and (**B**) the relative internalization. The values are expressed as mean ± SD of at least three biological replicates. The significant differences compared (ANOVA + Dunn’s; *p* < 0.05) are noted with asterisks (*).

To quantify the internalization of NFs in A549 cells, a colorimetric assay was conducted based on the reaction between digested free ferric ions and thiocyanate (see Supplementary Materials—Section S2). Due to its high toxicity, the Mn07 NFs were excluded from the study. For this evaluation, cells were exposed to increasing concentrations of NFs and incubated for 24 h. The data, shown in Figure 6A, indicate that the internalized Fe^3+^ quantity increased proportionally with the exposure dose of NFs. At the lowest concentration (15.625 µg/cm^2^), all three NF types showed approximately 30 µg/well of internalized Fe^3+^, while at the highest concentration (250 µg/cm^2^), the Fe^3+^ quantity increased approximately tenfold to the 300–400 µg/well, with slight variations depending on the NF type. For the first two doses, the internalized iron levels were comparable across all NF types. Among the three types of NFs, Mn05 displayed the best cellular internalization, with a statistical difference between them and the other two types of NFs at doses of 125 and 350 µg/cm^2^, respectively. At higher exposure doses, 125 and 250 µg/cm^2^, respectively, a saturation of internalization can be observed, which may be associated with a saturated active transport or a saturated intracellular compartment [48].

In terms of relative internalization (Figure 6B) (the ratio of the internalized amount to the exposure dose, expressed as a percentage—%), a decrease was noticed when increasing the exposure dose. At the lowest dose, it showed values between 70 and 85%, being statistically higher for Mn05. Similar to those observed at the lowest exposure dose, Mn05 nanoparticles showed higher internalization at all doses studied. Compared to Mn00 nanoparticles, at intermediate doses, about 20–40% higher internalization can be observed for Mn05. Higher internalization (intermediate between Mn00 and Mn05) could also be observed for Mn03. These results suggest the influence of the amount of Mn precursors used in the synthesis on the biological properties of the nanoparticles. In this case, the internalization increased with the increasing amount of Mn used in the synthesis. This higher internalization may be due to the smaller size of Mn03 and Mn05, the size being about two times smaller. Moreover, by increasing the amount of Mn used in the synthesis, the ferrimagnetic character of the NFs decreased, which may enhance both colloidal stability and cellular uptake. Mn00, characterized by stronger ferrimagnetic properties, tends to cluster and further hinder internalization by increasing particle size. In contrast, Mn03 and Mn05 NFs, with weaker ferrimagnetic properties and smaller diameters, exhibited reduced clustering and enhanced internalization efficiency.

**Figure 7:**
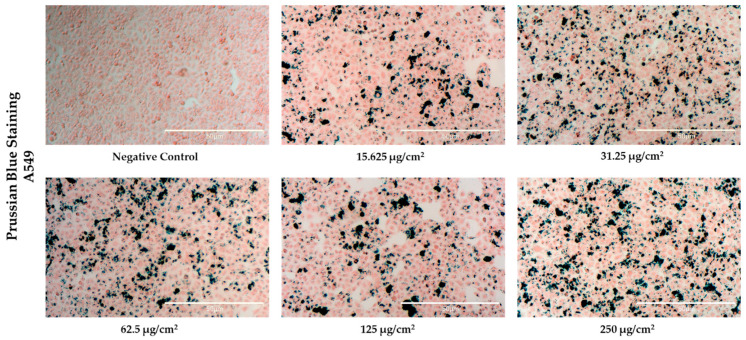
Microscopic images (magnification 100×) of Prussian Blue staining of A549 cells exposed for 24 h to Mn03 NFs at increased doses. The scale bar is 50 μm in all images.

The Mn03 NFs exhibited the best MH properties and an excellent toxicity profile across the tested dose range, making them the optimal choice for further in vitro MH studies. To assess their internalization, Prussian Blue staining was used to qualitatively evaluate iron content in A549 cells after 24 h of exposure. Microscopic images (Figure 7) revealed that Mn03 NFs (~118 nm) accumulated in the cytoplasm, primarily around the peri-nuclear region, confirming successful internalization by the A549 cells. This is in line with previous studies, which indicated that NPs ranging in size from 100 to 200 nm are easily internalized by malignant cells via numerous pathways [51,71]. The cells showed no significant cytotoxicity, maintaining their adherence and typical spherical morphology without signs of cellular volume shrinkage, regardless of the exposure dose. A study conducted by Liu et al. demonstrated that MCF-7 cells incubated with flower-like nanoparticles composed of iron and manganese (Fe_0.6_Mn_0.4_O_3_), which had sizes comparable to our synthesized NFs (102.7 ± 11 nm), exhibited no evidence of cytotoxicity [38]. The cells maintained their adherence to the substrate, preserved their typical shape, and displayed well-defined margins, supporting results consistent with our findings [38]. A closer examination of the images revealed a dose-dependent accumulation of Mn03 NFs, with dividing cells also visible. Notably, substantial amounts of Mn03 NFs were detected inside the cells even at a low dose of 15.62 µg/cm^2^.

### 3.6. In Vitro Magnetic Hyperthermia

The effect of AMF on cell integrity was evaluated as a prerequisite to conducting in vitro MH experiments. In our previous study, we demonstrated that a 30-min exposure of A549 cells to AMF did not result in a significant reduction in cell viability [48]. Similarly, Liu et al. performed this experiment using MCF-7 cells and observed comparable findings, reporting no significant decrease in cell viability after AMF exposure [38].

**Figure 8:**
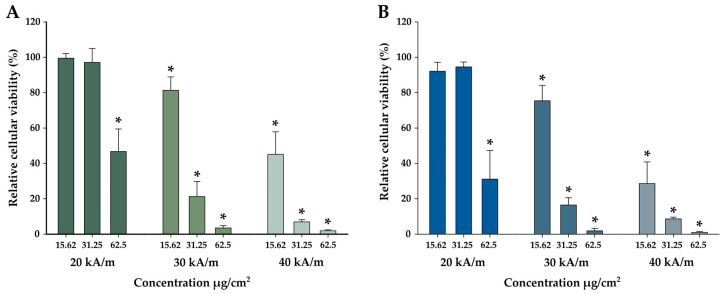
Cytotoxic effect of internalized Mn03 NFs in A549 cells evaluated after a 30 min exposure to AMF of 355 kHz and amplitudes of 20, 30, and 40 kA/m (**A**) and of internalized Mn03 NFs in A549 cells pre-exposed to a DC magnetic field of 65 kA/m for 30 s before performing MH in similar conditions (**B**). Cellular viability was measured using Alamar Blue assay and presented as the mean ± SD of five biological replicates. Data are presented as relative values to AMF negative control (100%). The significant differences compared to the negative control (ANOVA + Dunn’s; *p* < 0.05) are marked with asterisks (*).

Further, we conducted the in vitro MH study to evaluate the MH performance of Mn03 NFs at three magnetic field strengths (H = 20, 30, and 40 kA/m) and three concentrations (15.625, 31.25, and 62.5 µg/cm^2^) (Figure 8A). The first two H values (20 and 30 kA/m) remain within the new biological safety limit (~9.6 × 10^9^ A/ms), while the third value, 40 kA/m, exceeds this limit. At 20 kA/m, 30 min hyperthermic treatment had no impact on A549 cell viability at the two lower concentrations. The recorded sample temperatures were low, saturating at 39.2 °C and 41.0 °C, respectively (Supplementary Materials—Section S9). However, at 62.5 µg/cm^2^, cell viability dropped to 50% as the average temperature reached 42.5 °C. At this concentration, the iron levels within the cells doubled compared to the previous concentration (Figure 6A), indicating increased NF internalization into cancer cells, although microscopic images revealed uneven NF distribution, with clusters of varying sizes (Figure 7). At 30 kA/m, slight toxicity (80% cell viability, within the acceptable biological limit) was observed at 15.625 µg/cm^2^ and at 42.0 °C, but a significant decrease in viability (to 70%) occurred at 31.25 µg/cm^2^, where the average temperature rose to 44.2 °C. At the highest dose, the average temperature reached 49.2 °C, where the death of almost all cells was determined. At 40 kA/m, even the lowest dose reduced cell viability by half, with the recorded temperature hitting 44.5 °C. At the next two superior concentrations, the temperature significantly increased to 46.3 °C and 51.4 °C, respectively. This increase in temperature effectively brought cell viability to near zero.

In a second experiment, A549 cells incubated with NFs were pre-exposed to a static magnetic field of 65 kA/m for 30 s before MH treatment (Figure 8B). At 20 kA/m, pre-alignment had no effect on cell viability at the two lower concentrations, but it caused a significant drop (from 50% to 15%) at 62.5 µg/cm^2^, the difference in temperature being only 0.5 °C (Supplementary Materials—Section S9). A decline of 15% occurred at 30 kA/m for 31.25 µg/cm^2^ (0.7 °C temperature difference). At 40 kA/m, pre-alignment reduced viability by 20% even at the lowest concentration (15.625 µg/cm^2^), with almost no difference in registered temperature (0.2 °C). Across all three cases, we observed a slight average temperature increase (0.2–0.7 °C) between the two experimental sets.

A Three-Way ANOVA test with the exposure dose, AC field intensity, and (no) exposure to a static magnetic field as factors and the measured viability as the response was performed to evaluate the influence of each factor. In addition to the major impact of the exposure dose and intensity field on the measured viabilities, pre-exposure to a static magnetic field induced a statistical difference, with the pre-exposure step additionally decreasing the cellular viability after MH treatment. This is in accordance with the slight average temperature increase (0.2–0.7 °C) observed during the experiments.

The viability data of A459 cells plotted as a function of the saturation temperature reached during magnetic hyperthermia (Supplementary Materials—Section S10) for all concentrations and magnetic field strengths show a temperature T_0_ of 43.21 °C for reaching a viability of 50%, in agreement with our previous results for this cell line [65,72].

Minor temperature changes around this T_0_ temperature, where we notice a steep dependence of viability on temperature, could eventually explain the consistent decrease in cell viability caused by NFs’ pre-alignment in the static magnetic field. Nevertheless, mechanical effects may act alongside thermal effects to enhance cancer cell destruction. The mechanical effects may be caused by the alignment of the internalized Mn03 NFs determined by the static magnetic field applied before performing MH.

## 4. Conclusions

This study successfully synthesized and characterized Mn-doped ferrite nanoflowers (NFs) using the polyol synthesis method, highlighting their structural, magnetic, and functional properties for potential applications in magnetic hyperthermia (MH) therapy.

The incorporation of Mn into the magnetite matrix significantly altered the morphology from spherical nanoclusters to well-defined NFs, reducing particle size and size distribution. XRD analysis confirmed a single-phase inverse spinel structure across all samples, with lattice parameter expansion indicating successful Mn incorporation, without secondary phases. The Mn content influenced coercivity and blocking temperature, reducing dipolar interactions and softening the magnetic phase, which is favorable for biomedical applications.

The NFs demonstrated high specific absorption rate (SAR) values in both water and a solid PEG8k matrix under alternating magnetic fields (AMFs), with Mn03 showing superior heating efficiency. The exposure of A549 lung cancer cells to Mn03 NFs under MH conditions resulted in a temperature-dependent decrease in cell viability, with significant cell death observed at therapeutic temperatures above 43 °C. The pre-alignment of nanoparticles further improved MH efficiency, leading to increased cancer cell destruction.

Overall, this research underscores the promise of Mn-doped ferrite NFs as efficient and biocompatible heating agents for MH applications, with the potential for further development in cancer therapy. Future research should aim to optimize doping levels to achieve a balance between cytotoxicity and heating efficiency, assess long-term in vivo biocompatibility, and explore their potential for integrated therapies, including drug delivery and imaging-guided hyperthermia.

## Figures and Tables

**Table 1:** Size and crystal parameters of NFs.

Sample	TEM Diameter (nm)	Log Standard Deviation (nm)	XRD Diameter (nm)	Lattice Parameter (Å)
Mn00	222 ± 3	49 ± 4	33 ± 5	8.379
Mn03	120 ± 1	23 ± 2	21 ± 1	8.384
Mn05	118 ± 1	13 ± 1	23 ± 1	8.397
Mn07	112 ± 1	18 ± 2	24 ± 2	8.424

**Table 2:** Magnetic parameters of NFs.

Sample	4 K			300 K		
	M_s_ (emu/g)	H_c_ (mT)	M_r_ (emu/g)	M_s_ (emu/g)	H_c_ (mT)	M_r_ (emu/g)
Mn00	91.5	31.8	31.22	81.5	17.1	19.51
Mn03	93.1	29.5	28.67	81.0	15.8	15.44
Mn05	92.0	24.4	28.45	77.3	14.7	15.42
Mn07	88.5	23.5	27.62	75.3	12.6	14.74

## Data Availability

The original contributions presented in this study are included in the article/Supplementary Material. Further inquiries can be directed to the corresponding author.

## Supplementary Materials

The following supporting information can be downloaded at: https://www.mdpi.com/article/10.3390/pharmaceutics17030384/s1, Figure S1: Magnetic hyperthermia; Figure S2: Iron concentration determination calibration curve; Figure S3.1: Optical interference UV–Vis Spectra; Figure S3.2: Optical interference histograms; Figure S4: Magnets’ configuration for pre-alignment of the NFs; Figure S5: Hysteresis loops; Figure S6: Zero-field and field-cooled magnetization curves; Figure S7: Specific absorption rates of NFs; Figure S8: Biocompatibility data and the corresponding Hill function fitting curves; Table S1: Hill function parameters derived from the biocompatibility data; Figure S9: Heating curves of in vivo MH treatment; Figure S10: Cellular viability of A459 cells against the saturation temperature reached during MH.
