# Supplementary material for: Evaluating Manganese-Doped Magnetic Nanoflowers for Biocompatibility and In Vitro Magnetic Hyperthermia Efficacy
Source: Pharmaceutics. 2025 Mar 18;17(3):384. doi: 10.3390/pharmaceutics17030384 (PMC11944501; doi:10.3390/pharmaceutics17030384)
Supplement: Supplementary file 1 [file pharmaceutics-17-00384-s001.zip › pharmaceutics-3498701-supplementary.pdf]

Supplementary Materials

Evaluating Manganese-Doped Magnetic Nanoflowers for Biocompatibility and *in vitro* Magnetic Hyperthermia Efficacy

Andreea-Elena Petru [1,‡], Cristian Iacovita [2,‡], Ionel Fizesan [1], Roxana Dudric [3], Ionut-Valentin Crestin [1], Constantin Mihai Lucaciu [2,*], Felicia Loghin [1] and Bela Kiss [1]

[1] Department of Toxicology, Faculty of Pharmacy, "Iuliu Hațieganu" University of Medicine and Pharmacy, Pasteur, 6A, 400349 Cluj-Napoca, Romania; andreea.elen.petru@elearn.umfcluj.ro (A.-E.P.); ionel.fizesan@umfcluj.ro (I.F.); kbela@umfcluj.ro (K.B.); floghin@umfcluj.ro (F.L.); ionut.vale.crestin@elearn.umfcluj.ro (I.-V.C.).

[2] Department of Pharmaceutical Physics-Biophysics, Faculty of Pharmacy, "Iuliu Hatieganu" University of Medicine and Pharmacy, Pasteur 6, 400349 Cluj-Napoca, Romania; cristian.iacovita@umfcluj.ro (C.I.), clucaciu@umfcluj.ro (C.M.L.)

[3] Faculty of Physics, "Babes Bolyai" University, Kogalniceanu 1, 400084 Cluj-Napoca, Romania; roxana.pacurariu@phys.ubbcluj.ro (R.D.)

*Correspondence: clucaciu@umfcluj.ro (C.M.L)

‡ These authors contributed equally to the work.

S1. Magnetic hyperthermia :

The specific absorption rate (SAR) is defined as the heat released from a suspension of MNPs in unit time reported to the mass of iron content. It was used to quantify the heat performance of MNPs. For reliable determination of SAR, the temperature change ΔT versus time curves - where $\Delta T = T(t) - T_0$; T(t) is the temperature at time t and T_0 = 37 °C -, have been fitted with the Box-Lucas equation:

$$\Delta T = \frac{S_m}{k}\left(1 - e^{-k(t-t_0)}\right)$$

where the fitting parameters S_m and k are the initial slope of the heating curve and the constant describing the cooling rate, respectively. Thus, SAR can be calculated as:

$$\mathrm{SAR} = \frac{c\ m\, S_m}{m_{MNPs}}$$

where c is the specific heat of the colloid (in our case was approximated with the specific heat of water: $c = 4186\ J/kgK$ and PEG8K: $c = 2136\ J/kgK$ the MNPs contribution to the specific heat being negligible), $m = \rho/V$ is the mass of colloid, taken as the product between the density ($\rho_{water} = 0{,}997\ g/cm^3$ for water and $\rho_{PEG8K} = 1{,}125\ g/cm^3$ at 298K) and the volume (0.5 mL). Prior to each measurement the samples have been sonicated for 10 seconds to assure a good colloidal dispersion over the entire aqueous volume. Each SAR value is a mean of three measurements realized on three different samples.

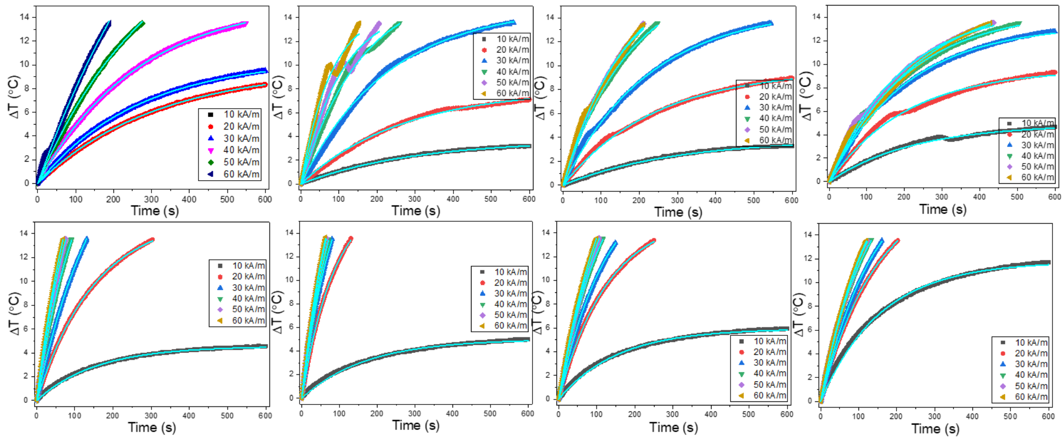

Figure S1. Groups of panels displaying the temperature change ΔT versus time curves fitted with Box-Lucas equation (blue curves) of NFs, dispersed in water (upper panels), and PEG8K (lower panels) at concentration of 1.00 mg_{NFs}/mL, recorded as a function of H (10 – 60 kA/m) at frequency of 355 kHz.

S2. Iron concentration determination calibration curve:

The iron content of NFs was measured using the Liebig reaction. In this regard, approximately 5 mg of NFs were magnetically separated and further mixed with a 10 mL of 12% HCl solution. The digestion was performed for 6 h at 80 °C, and the obtained solutions were centrifuged at 12,000 g for 10 mins to obtain the supernatants. The total Fe^{3+} content of supernatants (50 μL) was measured after an oxidation step with 1% ammonium persulfate (50 μL) by the reaction with 0.1 M potassium thiocyanate (100 μL) that yields a red-colored iron-thiocyanate complex. The absorbance was measured at λ = 490 nm using a Synergy 2 Multi-Mode Microplate Reader, and the Fe^{3+} content was calculated from a standard curve with concentrations ranging between 5 - 140 μg/mL (Fig. S1).

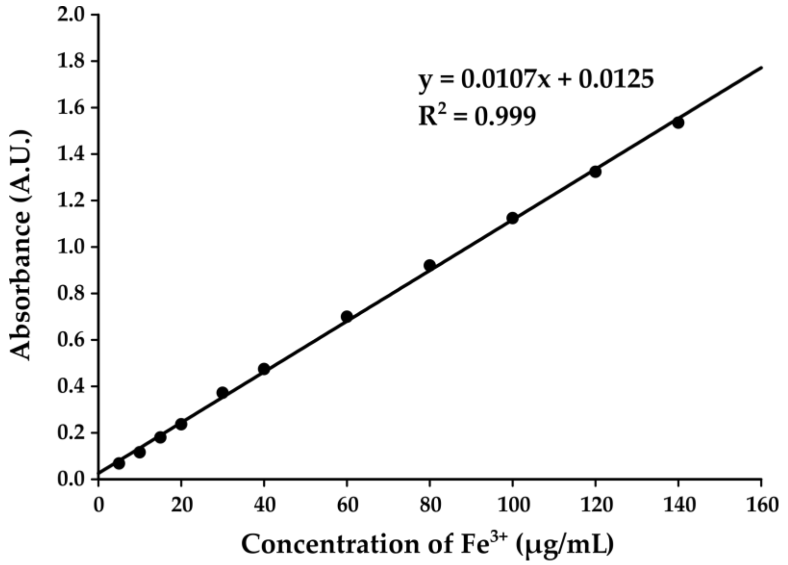

Figure S2. The absorbance of eleven standard Fe^{3+} colloidal solutions as a function on Fe^{3+} concentration measured at a λ = 490 nm. The values are expressed as mean ±SD of three replicates. The black line represents a linear regression of the experimental values.

S3. Optical interference:

Given the method employed to measure the iron content of NFs and that three of the four types of NFs that were synthesized were further doped with manganese, the issue was whether Mn, a transitional metal, could impede the Liebig Reaction. Thus, the Liebig reaction was performed in presence and in absence of Mn^{2+} ion. Briefly, solutions of Fe^{3+} with concentrations between 3.06 – 200 μg/mL and solutions of Mn^{2+} with concentrations between 25 – 400 μg/mL, were prepared. Standard curves for iron determination (3.06 – 200 μg/mL) were employed, as previously described in the "S2" section, in the presence of Mn^{2+} for each concentration (25 μg/mL, 50 μg/mL, 100 μg/mL, 200 μg/mL, 400 μg/mL). The red-colored iron-thiocyanate complex was quantified measuring the absorbance at λ = 490 nm using a Synergy 2 Multi-Mode Microplate Reader (Figure *S3.1.*).

Based on Figure *S3.2.*, no interference of Mn^{2+} was observed. The absorbance values were constant regardless of Mn^{2+} concentration, indicating that Mn^{2+} did not interfere and did not impede the reaction with Fe^{3+} ions.

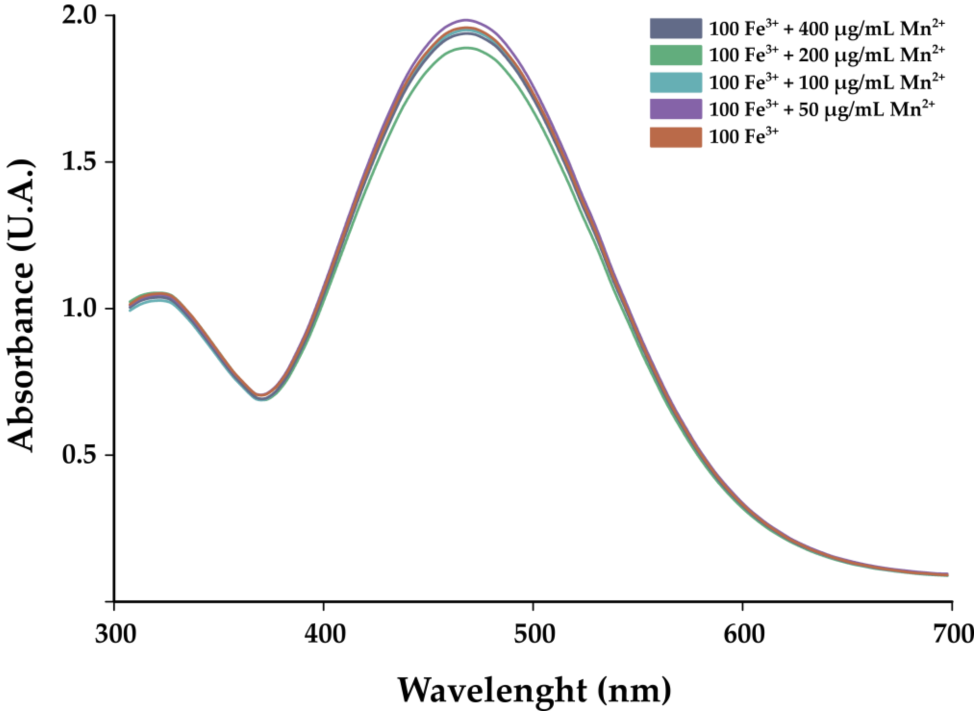

Figure S3.1. The UV-Vis spectra obtained for a standard with an Fe^{3+} concentration of 100 μg/mL in the presence of Mn^{2+} ions at concentrations ranging from 50 to 400 μg/mL

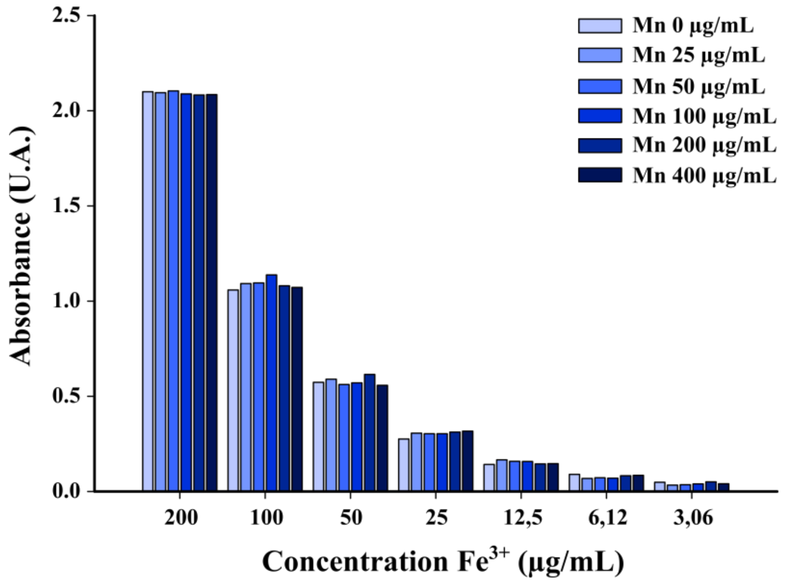

Figure S3.2. Evaluation of the optical interference of different concentrations of Mn^{2+} in the determination of iron content via Liebig Reaction. The results are expressed as mean values of three different replicates.

S4. Pre-alignment of the NFs dispersed in liquid PEG8K or internalized in A549 cells:

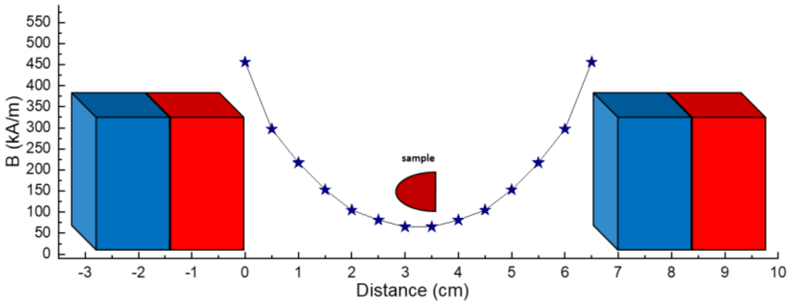

Figure S4. Schematic representation of setup used for the pre-alignment of the NFs either dispersed in liquid PEG8K at 80⁰C or internalized in A549 cells under a magnetic field of 65 kA/m, maximum attainable in MH experiments.

S5. Hysteresis loops of NFs:

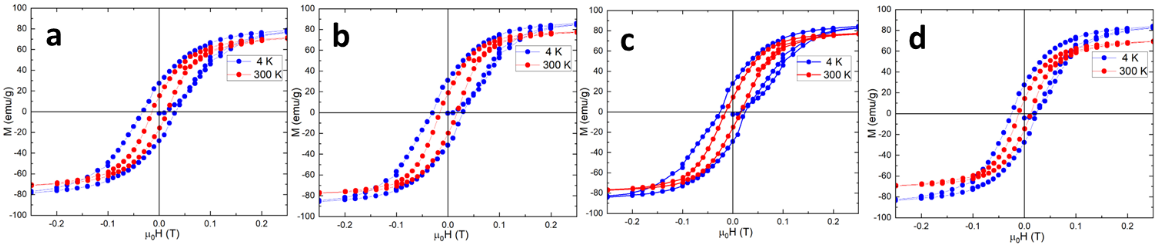

Figure S5. Zoom in hysteresis loops at both 4K and 300K for (**a**) Mn00, (**b**) Mn03, (**c**) Mn05 and (**d**) Mn07 MNPs.

S6. Zero-field and field-cooled magnetization curves:

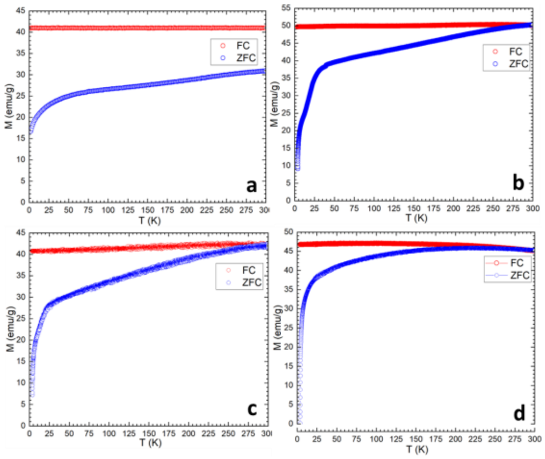

Figure S6. Zero-field-cooled (ZFC) and field-cooled (FC) magnetization curves for (**a**) Mn00, (**b**) Mn03, (**c**) Mn05 and (**d**) Mn07 MNPs.

S7. SAR of all four types of NFs dispersed in water and PEG8K:

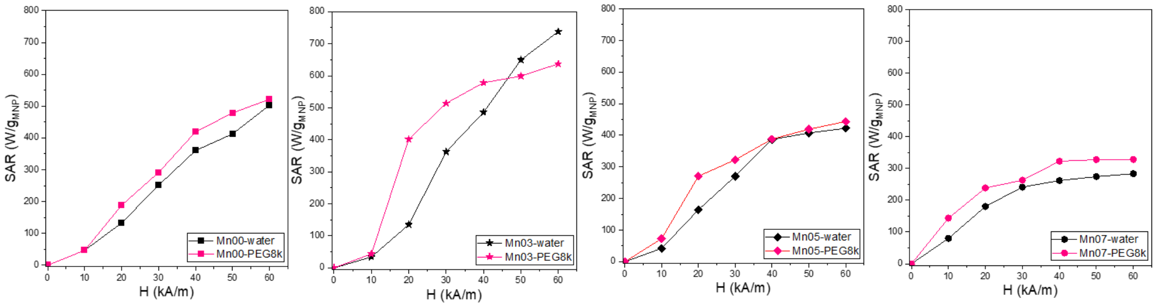

Figure S7. SAR values of NFs dispersed in water and pre-aligned in solid PEG8K.

S8. Biocompatibility data of NFs

The biocompatibility data obtained by using the Alamar Blue assay were fitted with the Hill function (OriginLab Pro 2016 software)

$$V=V_{start}+(V_{end}-V_{start})\ c^{n}/(k^{n}+c^{n})$$

where V is the viability, c the NFs concentration, k is the Michaelis constant, and n represents the Hill coefficient.

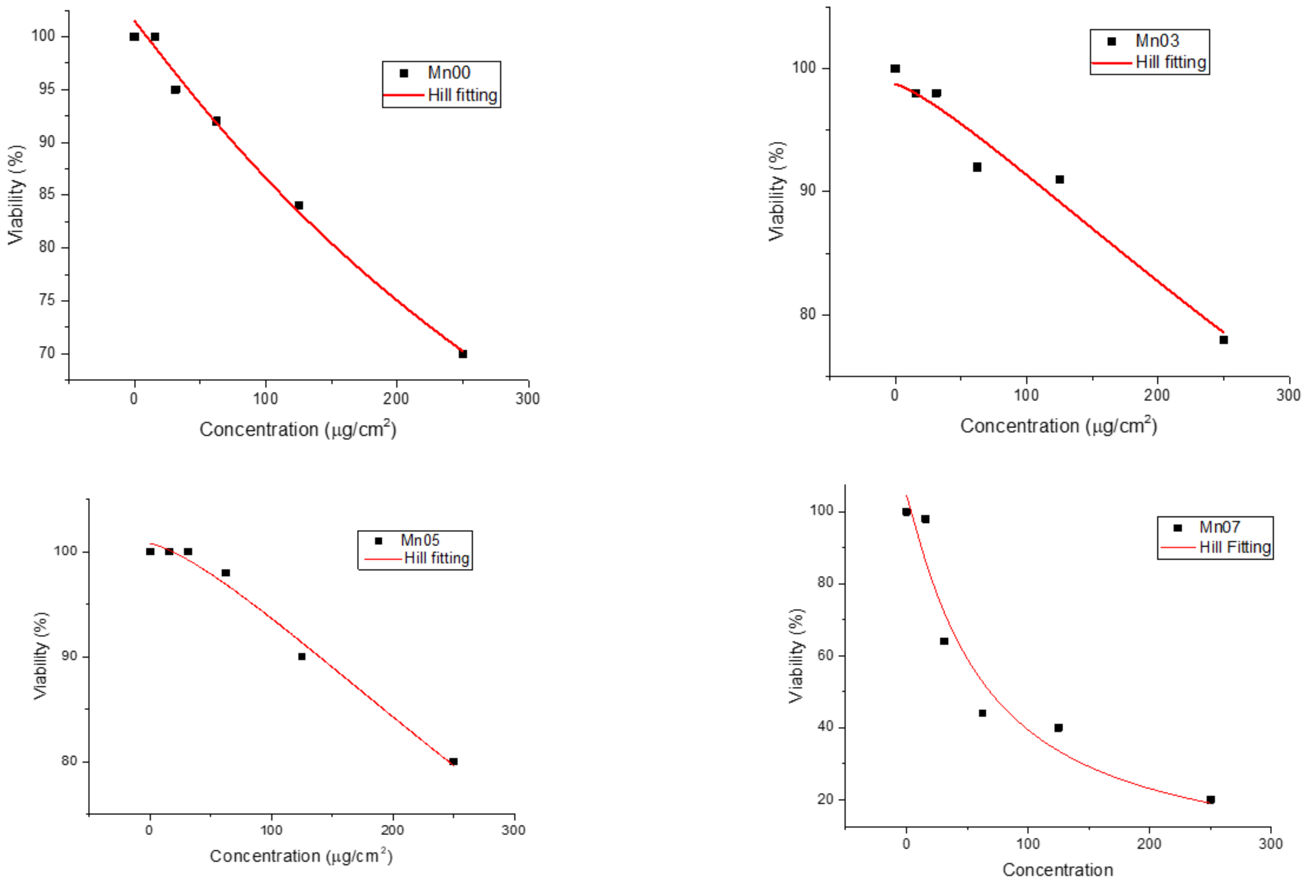

Figure S8. The cytocompatibility of NFs on the A549 cell line after a 24-hour incubation and the corresponding Hill function fitting curves.

Table S1 Hill function parameters derived from the biocompatibility data

NF type	LD50 (μg/cm²)	n
Mn00	546	1.0
Mn03	735	1.3
Mn05	660	1.2
Mn07	63	1.1

S9. Heating curves of *in vitro* MH treatment:

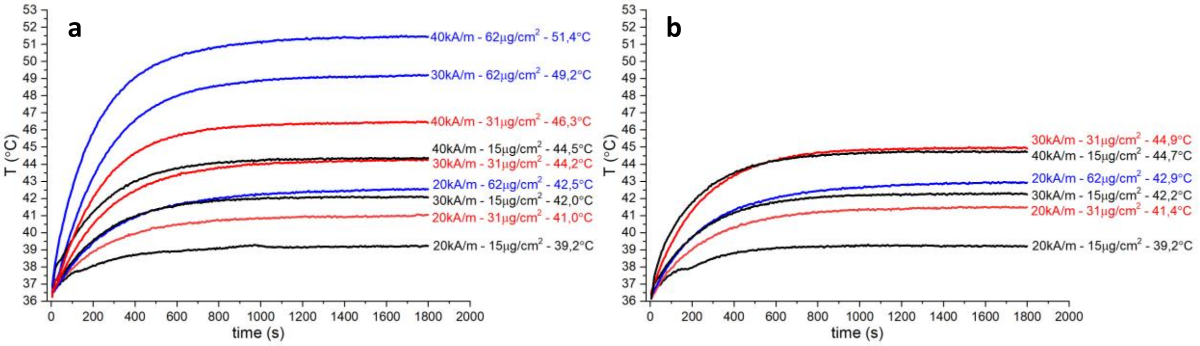

Figure S9. Heating curves of Mn03 NFs internalized in A549 in a volume of 300 µL at different H values (20, 30 and 40 kA/m) at a constant frequency of 355 kHz and different dosage level: 15 µg/cm² (black curves), 31 µg/cm² (red curves) and 62 µg/cm² (blue curves) in two different configurations: (a) randomly distributed in cells and (b) pre-aligned in a static magnetic field.

S10. Cellular viability versus saturation temperature:

The cellular viability, obtained using both biochemical assays, as a function of saturation temperature, reached during MH treatment, can be fitted with the following sigmoidal function:

$$C = \frac{A}{1 + e^{\frac{T - T_0}{dT}}}$$

where A represents the viability of control cells (100%), dT quantifies the temperature width for a given decrease in cell viability, and T_0 represents the temperature at which the viability reaches a value of 50%.

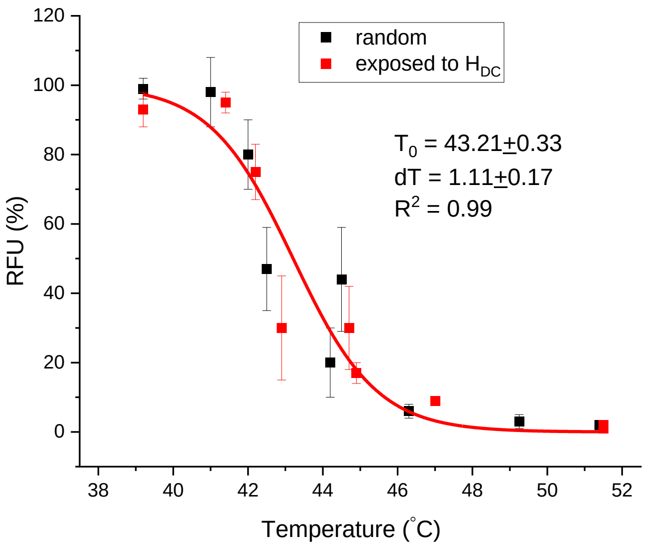

Figure S10. Cellular viability for A549 cells after MH with $Mn_{0.3}Fe_{2.6}O_4$ NFs, based on AB assay, plotted against the saturation temperatures reached during MH treatment and the corresponding fitting curve and fitting parameters based on equation 1.
